# Systems-based Discovery of Tomatidine as a Natural Small Molecule Inhibitor of Skeletal Muscle Atrophy*

**DOI:** 10.1074/jbc.M114.556241

**Published:** 2014-04-09

**Authors:** Michael C. Dyle, Scott M. Ebert, Daniel P. Cook, Steven D. Kunkel, Daniel K. Fox, Kale S. Bongers, Steven A. Bullard, Jason M. Dierdorff, Christopher M. Adams

**Affiliations:** From the ‡ Departments of Internal Medicine and Molecular Physiology and Biophysics, and Fraternal Order of Eagles Diabetes Research Center, Roy J. and Lucille A. Carver College of Medicine, University of Iowa, Iowa City, Iowa 52242 and; the § Iowa City Veterans Affairs Medical Center, Iowa City, Iowa 52246

**Keywords:** mTOR complex (mTORC), Muscle Atrophy, Skeletal Muscle, Skeletal Muscle Metabolism, Systems Biology, Tomatidine

## Abstract

Skeletal muscle atrophy is a common and debilitating condition that lacks an effective therapy. To address this problem, we used a systems-based discovery strategy to search for a small molecule whose mRNA expression signature negatively correlates to mRNA expression signatures of human skeletal muscle atrophy. This strategy identified a natural small molecule from tomato plants, tomatidine. Using cultured skeletal myotubes from both humans and mice, we found that tomatidine stimulated mTORC1 signaling and anabolism, leading to accumulation of protein and mitochondria, and ultimately, cell growth. Furthermore, in mice, tomatidine increased skeletal muscle mTORC1 signaling, reduced skeletal muscle atrophy, enhanced recovery from skeletal muscle atrophy, stimulated skeletal muscle hypertrophy, and increased strength and exercise capacity. Collectively, these results identify tomatidine as a novel small molecule inhibitor of muscle atrophy. Tomatidine may have utility as a therapeutic agent or lead compound for skeletal muscle atrophy.

## Teaser

**Background:** Skeletal muscle atrophy is a common and serious condition that lacks a pharmacologic therapy.

**Results:** We used a systems-based strategy to identify tomatidine, a natural compound from tomato plants, as a novel small molecule inhibitor of muscle atrophy.

**Conclusion:** Tomatidine may have utility as a therapeutic agent or lead compound for muscle atrophy.

**Significance:** These results suggest new therapeutic strategies for muscle atrophy.

## Introduction

Skeletal muscle atrophy is a common medical problem that can be caused by malnutrition, conditions of muscle disuse (*e.g.* limb casting, bed rest, osteoarthritis, stroke, amyotrophic lateral sclerosis, spinal cord injury, traumatic brain injury, and spaceflight), chronic disease (*e.g.* cancer, heart failure, COPD, renal failure, cirrhosis, HIV/AIDS, and rheumatoid arthritis), critical illness, endocrine disorders (*e.g.* diabetes, hypogonadism, and Cushing syndrome), and some medications (*e.g.* glucocorticoids, androgen antagonists, and cancer chemotherapeutic agents). Altogether, skeletal muscle atrophy is present in ∼13–24% of people over 60 years old and more than 50% of people over 80 years old; in the United States, this is ∼30 million people (1, 2).

In addition to being very common, muscle atrophy places tremendous burdens on patients, their families and society in general. Loss of strength and endurance from muscle atrophy limits activity, impairs quality of life, and leads to falls and fractures, as well as further muscle atrophy. In later stages, muscle atrophy causes debilitation and loss of independent living. In patients with orthopedic injuries, disuse muscle atrophy slows and often prevents full recovery (3). In patients with cancer, heart failure, COPD, or renal failure, muscle atrophy is an independent predictor of mortality (4). Muscle atrophy also affects the respiratory muscles, and in the ICU setting can delay recovery and impede weaning from mechanical ventilation (5). The estimated direct U.S. Healthcare costs of age-related muscle atrophy alone were $18.5 billion in 2000 and are much higher now (6).

Although skeletal muscle atrophy has broad clinical impact, current treatment recommendations (*e.g.* physical rehabilitation, nutritional optimization, and treatment of underlying disease) are often ineffective and/or unfeasible. Moreover, a pharmacologic therapy does not exist. Thus, skeletal muscle atrophy represents a very large unmet medical need. In a recent proof-of-concept study, we developed an unbiased, systems-based discovery method to identify small molecules that reduce skeletal muscle atrophy (7). In the current study, we used this method to identify a previously unrecognized small molecule inhibitor of skeletal muscle atrophy.

## EXPERIMENTAL PROCEDURES

### 

#### 

##### Chemicals and Antibodies

We obtained tomatidine from Enzo Life Sciences; rapamycin from Sigma; and [^3^H]tyrosine (40 Ci/mmol) from American Radiolabeled Chemicals. Anti-troponin (CT3), anti-myosin heavy chain (MyHC) I (BA-F8), anti-MyHC IIa (SC-71), anti-MyHC IIb (BF-F3), and anti-MyHC IIx (6H1) mouse monoclonal antibodies were obtained from the University of Iowa Developmental Studies Hybridoma Bank. Alexa Fluor 488-conjugated anti-mouse IgG_1_, Alexa Fluor 350-conjugated anti-mouse IgG_2b_ and Alexa Fluor 555-conjugated anti-mouse IgM were obtained from Invitrogen. Anti-PGC-1α1 rabbit polyclonal antibody (ab54481) was obtained from Abcam. All other antibodies were obtained from Cell Signaling Technologies (anti-S6K (2308S), anti-phospho-S6K (Thr-389) (9234S), anti-Akt (4691S), anti-phospho-Akt (Ser-473) (4060S), anti-α-tubulin (2125S), and horseradish peroxidase (HRP)-conjugated anti-rabbit IgG (7074S)).

##### Tissue Culture Media

Medium A is SkBM-2 Basal Media (Lonza CC-3246) supplemented with BulletKit (Lonza CC-3245). Medium B is DMEM/F-12 (Invitrogen 11320-033) containing antibiotics (100 units/ml penicillin and 100 μg/ml streptomycin sulfate) and 2% (*v*/*v*) horse serum (HS; Hyclone SH3007403). Medium C is high glucose Dulbecco's Modified Eagle's Medium (DMEM) (Invitrogen 11965-092) containing antibiotics and 10% (*v*/*v*) fetal bovine serum (HyClone SH30071.03). Medium D is DMEM containing antibiotics and 2% (*v*/*v*) HS.

##### Culture, Differentiation, and Treatment of Human Skeletal Myotubes

Human skeletal muscle myoblast cells (Lonza CC-2580) were maintained in medium A at 37 °C and 5% CO_2_, and used up to passage 7. On day 0, myoblasts were set up for experiments in 6-well plates in medium A at 1 × 10^5^ cells per well. On day 2, myoblast differentiation was induced by replacing medium A with medium B. On day 7, myotubes were rinsed with phosphate-buffered saline (PBS), medium B was replaced with medium A, and vehicle (0.1% (*v*/*v*) DMSO) or tomatidine was directly added to the media. Myotubes were then incubated for 48 h before quantification of total cellular protein, mitochondrial DNA, and myotube size. In all myotube experiments, tomatidine was prepared as a 1 mm stock solution in DMSO.

##### Culture, Differentiation, and Treatment of Mouse C2C12 Skeletal Myotubes

Mouse C2C12 myoblasts (ATCC CRL-1772) were maintained at 37 °C and 5% CO_2_ in medium C and used up to passage 7. On day 0, myoblasts were set up for experiments in 6-well plates in medium C at 2.5 × 10^5^ cells per well. On day 1, myoblast differentiation was induced by replacing medium C with medium D. On day 7, myotubes were rinsed with PBS and medium D was replaced with medium C. In assays of protein phosphorylation and mRNA expression, vehicle or rapamycin were immediately added to the media; vehicle or tomatidine were added 4 h later, and myotubes were harvested 1 h later (for analysis of protein phosphorylation) or 2 h later (for analysis of mRNA expression). In other assays, vehicle, tomatidine and/or rapamycin were immediately added to the media and myotubes were harvested 30 h later (for analysis of protein synthesis) or 48 h later (for analysis of total cellular protein, mitochondrial DNA and myotube size).

##### Quantification of Total Cellular Protein and Mitochondrial DNA

To quantify myotube total cellular protein, we used the Illustra TriplePrep Kit (GE Healthcare). In each sample, the amount of protein was normalized to the amount of DNA, which was not altered by tomatidine. To quantify total cellular protein in skeletal muscle, we used the BCA Kit (Pierce). To quantify mitochondrial DNA, we used the DNeasy Blood and Tissue Kit (Qiagen) to isolate total DNA from myotubes and muscle, and then determined the amount of mitochondrial and nuclear DNA by quantitative real-time PCR (qPCR) according to previously described methods (8).

##### Quantification of Myotube Size

Myotubes were rinsed once with PBS, fixed for 15 min in 4% paraformaldehyde, permeabilized for 2 min in a 1:1 mixture of methanol and acetone, blocked for 1 h at 25 °C in 5% normal goat serum (NGS; Sigma), and then incubated for 16 h at 4 °C in a 1:250 dilution of anti-troponin antibody in 5% NGS. Myotubes were then rinsed with PBS, incubated for 1 h at 25 °C in a 1:1000 dilution of Alexa 488-conjugated anti-mouse IgG, rinsed again with PBS, covered in Vectashield mounting medium, and imaged with an Olympus IX-71 microscope equipped with a DP-70 camera and epifluorescence filters. Image analysis was performed using ImageJ software and the diameter of each myotube was determined by averaging three width measurements per myotube.

##### Immunoblot Analysis

Protein extracts from myotubes and mouse skeletal muscle were prepared as previously described (8, 9). An aliquot of each protein extract was then mixed with 0.25 volume of sample buffer (250 mm Tris-HCl, pH 6.8, 10% SDS, 25% glycerol, 0.2% (w/v) bromphenol blue, and 5% (w/v) 2-mercaptoethanol) and heated for 5 min at 95 °C. A separate aliquot of each extract was used to determine protein concentration by the BCA kit, after which an equivalent amount of protein from each sample was subjected to SDS-PAGE, and then transferred to 0.45 nitrocellulose membranes (Bio-Rad 162-0115). Immunoblots were performed at 4 °C for 16 h using a 1:2000 dilution of primary antibodies. Bound antibodies were visualized by chemiluminescence (SuperSignal West Pico; Thermo Scientific) using a 1:2000 dilution of HRP-conjugated anti-rabbit IgG.

##### Quantification of Protein Synthesis

[^3^H]Tyrosine (4 μCi/ml) was directly added to the medium, and myotubes were incubated for 30 min at 37 °C. Control dishes of myotubes were incubated at 4 °C, which inhibits protein synthesis but not nonspecific background incorporation of [^3^H]tyrosine. Following incubation, myotubes were washed three times with Hank's balanced salt solution (Invitrogen, 14175095) supplemented with 2 mm non-radioactive l-tyrosine (Sigma). Myotubes were then fixed with 10% trichloroacetic acid (TCA) for 10 min on ice, and then scraped and collected into microfuge tubes. Samples were then incubated at 4 °C for 1 h before centrifugation at 15,000 × *g* for 15 min at 4 °C to pellet acid-insoluble protein. Pellets were washed once with 10% TCA and then dissolved in 1 N NaOH containing 1% sodium deoxycholate. An aliquot was taken to quantify protein concentration by the BCA kit (Pierce), and another aliquot was neutralized with 8 N HCl and placed in scintillation mixture for measurement of acid-insoluble radioactivity. The final results were obtained by subtracting the background counts obtained from dishes incubated at 4 °C, then normalizing the specific counts to the total mg of protein per well under each condition.

##### Quantification of mRNA Levels in Myotubes and Muscle

RNA was extracted from myotubes and muscle, treated with DNase, and analyzed with quantitative real-time RT-PCR according to previously described methods (7, 8).

##### Mouse Protocols

Male C57BL/6 mice were obtained from the National Cancer Institute at age 6–8 weeks and housed in colony cages at 21 °C with 12 h light/12 h dark cycles. Unless otherwise indicated, mice were maintained on standard chow (Harlan Teklad formula 7013). Tomatidine was custom added to chow formula 7013 by Harlan Teklad. In all mouse studies, age- and weight-matched mice were randomly assigned to control or treatment groups, and investigators who obtained the results were blinded to the intervention. Forelimb grip strength was determined using a triangular pull bar attached to a grip strength meter (Columbus Instruments). Each mouse was subjected to 5 consecutive tests to obtain the peak value, as described previously (7, 10). Mouse exercise capacity was determined as described previously (10): for 2 days, mice were acclimated to running on a motor-driven open treadmill with a shock grid (Columbus Instruments) for 5 min/day. During acclimation, the treadmill speed was set at 14 m/min and the treadmill incline was set at 0%. On the third day, exercise tolerance was tested: the shock grid was set at 0.2 mA and the treadmill incline was set at 10%. For the first 5 min of testing, treadmill speed was set at 10 m/min. Every 2 min thereafter, the treadmill speed was increased by 2 m/min. Running was terminated when mice contacted the shock grid for 10 s. Body composition measurements were obtained using a Bruker Minispec LF 90_II_. For intraperitoneal injections, tomatidine was suspended in corn oil at a concentration of 2.5 mg/ml, vortexed for 5 min, immediately loaded into 1 ml syringes, and then injected intraperitoneal at a volume of 10 μl/g body weight. Mice were fasted for 24 h by removing food but not water. Unilateral hindlimb immobilization was performed under isoflurane anesthesia using an Autosuture Royal 35W skinstapler (Tyco Healthcare) as described previously (8, 11, 12). Immobilized hindlimbs were remobilized by removing the metal clip under isoflurane anesthesia. All animal procedures were approved by the Institutional Animal Care and Use Committee of the University of Iowa.

##### Histological Analysis of Skeletal Muscle Fibers and Adipocytes

Harvested skeletal muscles were immediately embedded in T.F.M. compound (Tissue-Tek) and snap frozen using a Stand-Alone Gentle Jane (Instrumedics Inc.). We then prepared 10 μm sections from the muscle mid-belly using a Microm HM 505E cryostat. Hematoxylin and eosin (H&E) stains were performed using a DRS-601 automatic slide stainer (Sakura) following fixation in ice cold zinc formalin for 15 min. Fiber type-specific stains were performed as described previously (13). Briefly, cryosections were blocked for 1 h at 25 °C in 10% NGS, and then incubated for 2 h at 25 °C in 10% NGS containing a 1:50 dilution of anti-MyHC I antibody, a 1:600 dilution of anti-MyHC IIa antibody, a 1:100 dilution of anti-MyHC IIb antibody and a 1:50 dilution of anti-MyHC IIx antibody. Sections were then rinsed with PBS, incubated for 1 h at 25 °C in 10% NGS containing a 1:500 dilution of Alexa Fluor 488-conjugated anti-mouse IgG_1_, Alexa Fluor 350-conjugated anti-mouse IgG_2b_ and Alexa Fluor 555-conjugated anti-mouse IgM, and mounted in Prolong Gold (Invitrogen). Mouse adipose tissue was fixed in 10% neutral buffered formalin and embedded in paraffin. We then prepared 4 μm sections using a Microm HM 355S microtome and stained the sections with H&E. All histological sections were examined and photographed using an Olympus BX-61 automated upright microscope equipped with CellSens digital imaging software. Image analysis was performed using ImageJ software. Skeletal muscle fiber size was analyzed by measuring the lesser diameter (minimal *Feret* diameter) of muscle fibers, as recommended elsewhere (14).

##### Analysis of Skeletal Muscle Specific Force

Mice were euthanized and the lower hindlimb was removed by transecting the upper hindlimb mid-way through the femur. The lower hindlimb was immediately placed in Krebs Ringer solution (120 mm NaCl, 23.8 mm NaHCO_3_, 10 mm d-glucose, 4.8 mm KCl, 2.5 mm CaCl_2_, 1.2 mm KH_2_PO_4_, 1.2 mm MgSO_4_, 5 mm HEPES, 2.5 mm CaCl_2_), and then aerated with 95% O_2_ and 5% CO_2_. The gastrocnemius, soleus, TA muscles, as well as the distal half of the tibia and fibula, were removed, leaving the intact EDL muscle. A staple with an attached suture was placed through the knee joint, and the preparation was mounted vertically in a water jacket bath (Aurora Scientific 1200A Intact Muscle Test System, filled with aerated Krebs-Ringer solution (95% O_2_, 5% CO_2_ at 25 °C)) by attaching the suture to a servocontrolled lever (Model 805A; Aurora Scientific) and clamping the metatarsals inferiorly. Isometric contractile properties of the EDL muscle were evaluated according to methods previously described (7). To produce a maximum isometric contraction, muscles were field stimulated with supramaximal square-wave pulses (0.2 ms duration) delivered to two platinum plate electrodes flanking the length of the muscle. Optimum muscle length (L_o_) and optimum stimulation voltage were determined by micromanipulating muscle length and eliciting contractions until the peak potentiated state was reached. Maximum isometric tetanic force (P_o_) was determined from the plateau of the tetanic curve following stimulation with supramaximal voltage (40 V) at 150 Hz with 2 min rest between recordings to prevent fatigue. Contractile measurements were recorded using a digital controller (Model 600A; Aurora Scientific) operating ASI Dynamic Muscle Control acquisition software (v4.1, Aurora Scientific). Following force testing, muscles were removed from the bath, trimmed of tendons, and weighed on an analytical balance. Optimum fiber length (L_f_) was determined by multiplying L_o_ by a previously determined ratio of fiber length: muscle length (0.44 for the EDL muscle (15)). Muscle cross sectional area was determined by dividing muscle weight by the product of L_f_ and 1.06 mg/mm^3^ (the density of mammalian skeletal muscle (16)). Muscle mass, L_f_ and P_o_ were then be used to calculate maximum tetanic force normalized to cross-sectional area (specific force).

##### Statistical Analysis

Comparisons between two groups used unpaired Student's *t* tests. Comparisons involving repeated measurements from different statistical units were analyzed by one-way ANOVA with Dunnett's or Tukey's post hoc tests.

## RESULTS

### 

#### 

##### mRNA Expression Signatures of Tomatidine Negatively Correlate to mRNA Expression Signatures of Skeletal Muscle Atrophy

**FIGURE 1.**
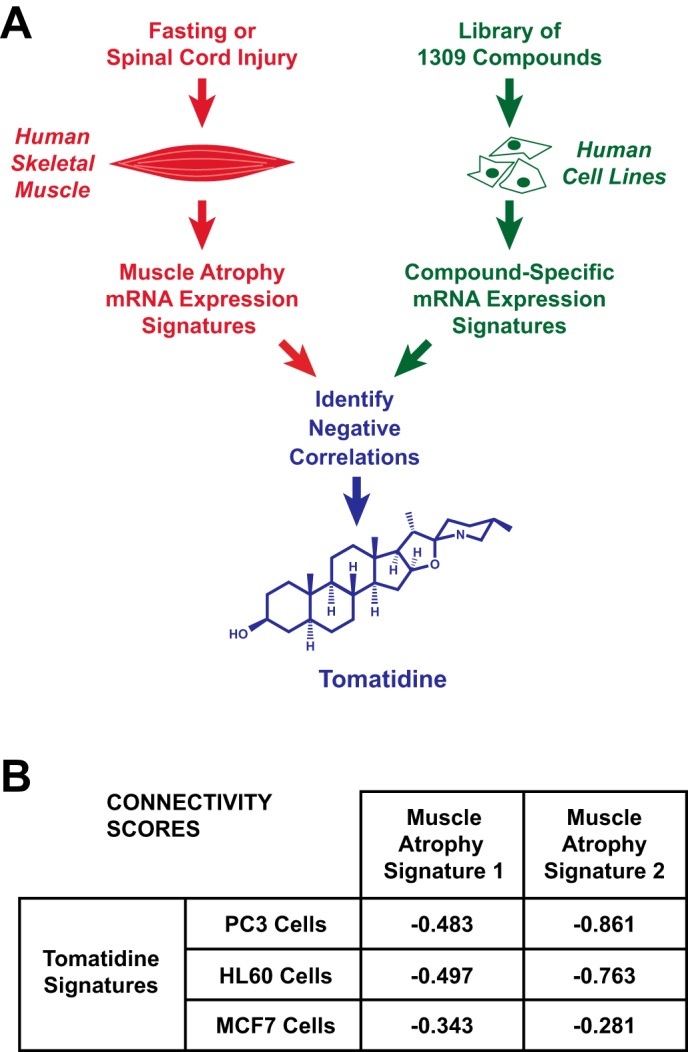
**Identification of tomatidine as a candidate small molecule inhibitor of skeletal muscle atrophy.** *A*, systems-based discovery strategy: we compared two mRNA expression signatures of skeletal muscle atrophy (muscle atrophy signatures 1 and 2) to mRNA expression signatures of 1309 small molecules in the Connectivity Map, searching for negative correlations. *B*, in three human cell lines (PC3, HL60, and MCF7), the mRNA expression signatures of tomatidine negatively correlated to both muscle atrophy signatures. Data are connectivity scores, which indicate the strength of the negative correlation on a scale from 0 to −1.

mRNA signatures are patterns of positive and negative changes in mRNA levels that occur in response to perturbations such as a disease or small molecule. In a previous study, we identified two genome-wide mRNA expression signatures of skeletal muscle atrophy (7). Muscle atrophy signature 1 consists of mRNAs that are similarly altered by fasting in both human and mouse skeletal muscle (7). Muscle atrophy signature 2 consists of mRNAs that are similarly altered by fasting and spinal cord injury in human skeletal muscle (7). Because changes in skeletal muscle gene expression play a crucial role in the pathogenesis of skeletal muscle atrophy (8, 17, 18), we hypothesized that we might discover a pharmacologic inhibitor of skeletal muscle atrophy by identifying a small molecule whose mRNA expression signature negatively correlates to muscle atrophy signatures 1 and 2. To identify such a small molecule, we used the Connectivity Map (19) to compare muscle atrophy signatures 1 and 2 to the mRNA expression signatures of 1309 small molecules in several human cancer cell lines (Fig. 1*A*). Interestingly, in three human cell lines, the mRNA signature of tomatidine negatively correlated to muscle atrophy signatures 1 and 2 (Fig. 1*B*).

Tomatidine is a steroidal alkaloid and the aglycone of α-tomatine, an abundant glycoalkaloid in tomato plants that mediates plant defense against fungi, bacteria, viruses, and predatory insects (20). When consumed by animals, α-tomatine is hydrolyzed by stomach acid and intestinal bacteria to tomatidine, which is absorbed by the gut (20). Tomatidine's effects on skeletal muscle are unknown. However, the finding that the mRNA expression signature of tomatidine negatively correlated to signatures of muscle atrophy suggested that tomatidine might have an anti-atrophic (anabolic) effect in skeletal muscle.

##### Tomatidine Stimulates Hypertrophy of Cultured Skeletal Myotubes from Humans and Mice

**FIGURE 2.**
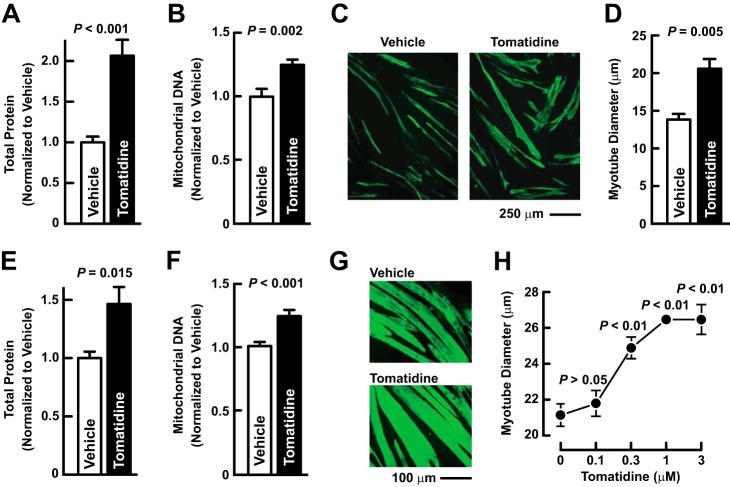
**Tomatidine stimulates anabolism in cultured skeletal myotubes from humans and mice.** *A–D*, human skeletal myotubes were incubated with either vehicle (0.1% DMSO) or 1 μm tomatidine for 48 h. *A*, total cellular protein. Data are means ± S.E. from 9 samples per condition. *B*, mitochondrial DNA. Data are means ± S.E. from 9 samples per condition. *C*, representative immunofluorescence images of myotubes stained with anti-troponin antibody. *D*, quantification of myotube size. Data are means ± S.E. from three experiments, each with > 300 myotubes measured per condition. *E–G*, mouse C2C12 skeletal myotubes were incubated with either vehicle (0.1% DMSO) or 1 μm tomatidine for 48 h. *E*, total cellular protein. Data are means ± S.E. from four samples per condition. *F*, mitochondrial DNA. Data are means ± S.E. from 15 samples per condition. *G*, representative immunofluorescence images of myotubes stained with anti-troponin antibody. *H*, mouse C2C12 skeletal myotubes were incubated with the indicated concentration of tomatidine for 48 h before quantification of myotube size. Data are means ± S.E. from three experiments, each with > 250 myotubes measured per condition. *A*, *B*, *D–F*, *H*, *p* values were determined with Student's *t* test (*A*, *B*, *D–F*) or ANOVA with Dunnett's post hoc test (*H*).

To test the hypothesis that tomatidine stimulates skeletal muscle anabolism, we investigated tomatidine's effects on three anabolic processes (accumulation of total cellular protein, accumulation of mitochondrial DNA, and cell growth) in cultured skeletal myotubes from two species (humans and mice). Importantly, the myotubes were terminally differentiated and thus post-mitotic. In primary human myotubes, a 48 h incubation with 1 μm tomatidine increased total cellular protein (Fig. 2*A*), increased mitochondrial DNA (Fig. 2*B*), and stimulated myotube hypertrophy (Fig. 2, *C* and *D*). Similarly, in mouse C2C12 myotubes, a 48 h incubation with 1 μm tomatidine increased total cellular protein (Fig. 2*E*), increased mitochondrial DNA (Fig. 2*F*), and stimulated myotube hypertrophy (Fig. 2, *G* and *H*). Dose-response experiments indicated that the EC_50_ for tomatidine was < 300 nm (Fig. 2*H*). These data indicated that tomatidine stimulates anabolism in skeletal muscle cells from both humans and mice.

##### Tomatidine Stimulates Muscle Cell Growth by Activating mTORC1 Signaling

Activation of mammalian target of rapamycin complex 1 (mTORC1)^2^ is a key event in skeletal muscle anabolism (21). mTORC1 signaling stimulates skeletal muscle hypertrophy and reduces skeletal muscle atrophy by several mechanisms including increased protein synthesis, increased mitochondrial biogenesis, and increased expression of anabolic genes encoding insulin-like growth factor-1 (IGF-1) and peroxisome proliferator-activated receptor-γ coactivator-1α1 (PGC-1α1) (21–26). Based on these considerations, we hypothesized that tomatidine might stimulate skeletal muscle anabolism by activating mTORC1 signaling.

**FIGURE 3.**
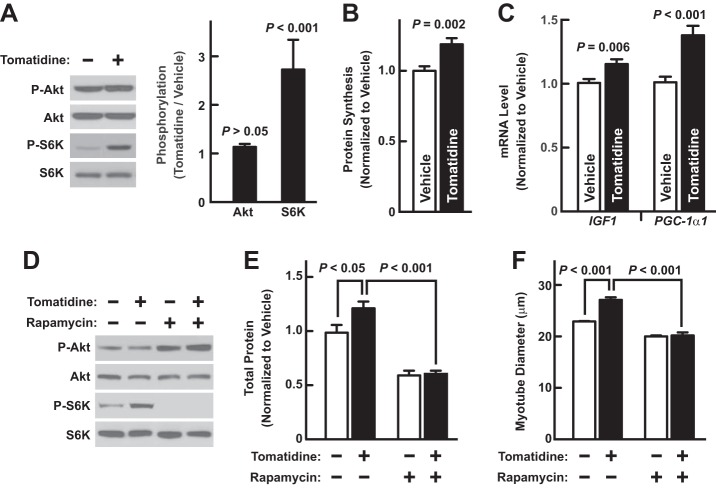
**Tomatidine stimulates anabolism by activating mTORC1 signaling.** *A*, C2C12 skeletal myotubes were incubated with vehicle (0.1% DMSO) or 1 μm tomatidine for 1 h before immunoblot analysis with the indicated antibodies. *Left*, representative immunoblots. *Right*, quantification. Data are means ± S.E. from nine independent experiments. *B*, C2C12 myotubes were incubated with vehicle or 1 μm tomatidine for 30 h before measurement of protein synthesis. Data are means ± S.E. from 14 samples per condition. *C*, C2C12 myotubes were incubated with vehicle or 1 μm tomatidine for 2 h before qPCR analysis of *IGF1* and *PGC-1*α*1* mRNA levels. Data are means ± S.E. from 6 samples per condition. *D*, C2C12 myotubes were incubated for 1 h with vehicle or 1 μm tomatidine in the absence or presence of 100 nm rapamycin before immunoblot analysis with the indicated antibodies. *E* and *F*, C2C12 myotubes were incubated for 48 h with vehicle or 1 μm tomatidine in the absence or presence of 100 nm rapamycin before quantification of total cellular protein (*E*) and myotube size (*F*). *E*, data are means ± S.E. from 8 samples per condition. *F*, data are means ± S.E. from three experiments, each with > 150 myotube measurements per condition. *A–C*, *E–F*, *p* values were determined with Student's *t* test (*A–C*) or ANOVA with Tukey's post hoc test (*E–F*).

To test this hypothesis, we incubated C2C12 myotubes with 1 μm tomatidine for 1 h. We found that tomatidine increased phosphorylation of a key mTORC1 substrate, S6 kinase (S6K) (Fig. 3*A*). In addition, tomatidine increased protein synthesis (Fig. 3*B*) and *IGF1* and *PGC-1*α*1* mRNAs (Fig. 3*C*). To determine if mTORC1 signaling is required for tomatidine-mediated cell growth, we tested the effect of an mTORC1 inhibitor, rapamycin. As expected, rapamycin abolished the effect of tomatidine on S6K phosphorylation (Fig. 3*D*). Moreover, rapamycin inhibited tomatidine-mediated protein accretion and myotube growth (Fig. 3, *E* and *F*). Collectively, these data indicate that tomatidine stimulates muscle cell growth by activating mTORC1 signaling.

We also examined the effect of tomatidine on phosphorylation (activation) of Akt, an anabolic kinase that can contribute to mTORC1 activation. We found that tomatidine did not increase Akt phosphorylation (Fig. 3*A*). In addition, rapamycin did not inhibit Akt phosphorylation, consistent with specificity for mTORC1 (Fig. 3*D*).

##### Tomatidine Stimulates mTORC1 Activity and Skeletal Muscle Hypertrophy In Vivo, Leading to Increased Strength and Exercise Capacity

**FIGURE 4.**
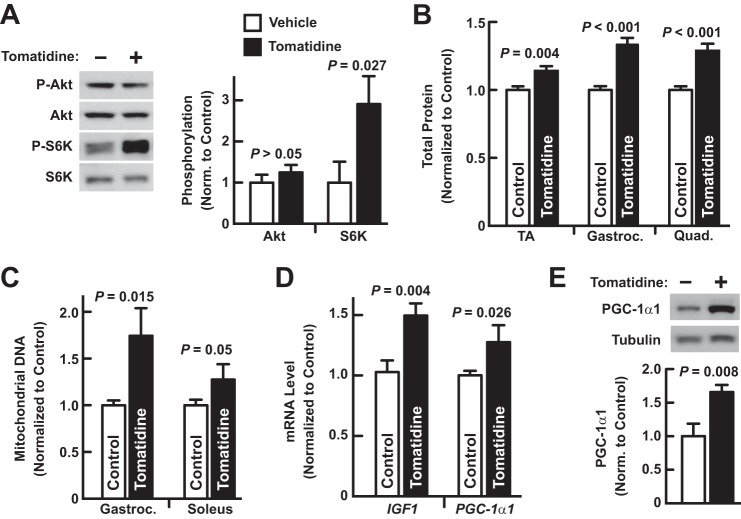
**Tomatidine stimulates mTORC1 activity in mouse skeletal muscle.** Seven-week-old mice were provided *ad libitum* access to either standard chow (control) or standard chow supplemented with 0.05% tomatidine for 5 weeks before skeletal muscles were harvested for further analysis. *A*, immunoblot analysis of total and phosphorylated Akt and S6K. *Left*, representative immunoblots. *Right*, quantification. Data are means ± S.E. from 5 gastrocnemius muscles per condition. *B*, total cellular protein in the TA, gastrocnemius (*gastroc*.) and quadriceps (*quad*.) muscles. Data are means ± S.E. from 6–7 muscles per condition. *C*, mitochondrial DNA in the gastroc. and soleus muscles. Data are means ± S.E. from 6–8 muscles per condition. *D*, qPCR analysis of *IGF1* and *PGC-1*α*1* mRNA levels. Data are means ± S.E. from 5–7 TA muscles per condition. *E*, immunoblot analysis of PGC-1α1. *Upper*, representative immunoblots; α-tubulin served as a loading control. *Lower*, quantification. Data are means ± S.E. from 5 TA muscles per condition. *A–E*, *p* values were determined with Student's *t* test.

To test the hypothesis that tomatidine stimulates skeletal muscle anabolism *in vivo*, we provided young mice (7 weeks old) *ad libitum* access to standard chow that was supplemented with 0.05% (w/w) tomatidine. Five weeks later, we examined the effect of tomatidine on skeletal muscle. As it had done in cultured myotubes, tomatidine increased mTORC1 activity, as evidenced by increased S6K phosphorylation (Fig. 4*A*), increased total cellular protein (Fig. 4*B*), increased mitochondrial DNA (Fig. 4*C*), increased *IGF1* and *PGC-1*α*1* mRNAs (Fig. 4*D*), and increased PGC-1α1 protein (Fig. 4*E*). In contrast to its effect on mTORC1, tomatidine did not increase phosphorylation of Akt (Fig. 4*A*).

**FIGURE 5.**
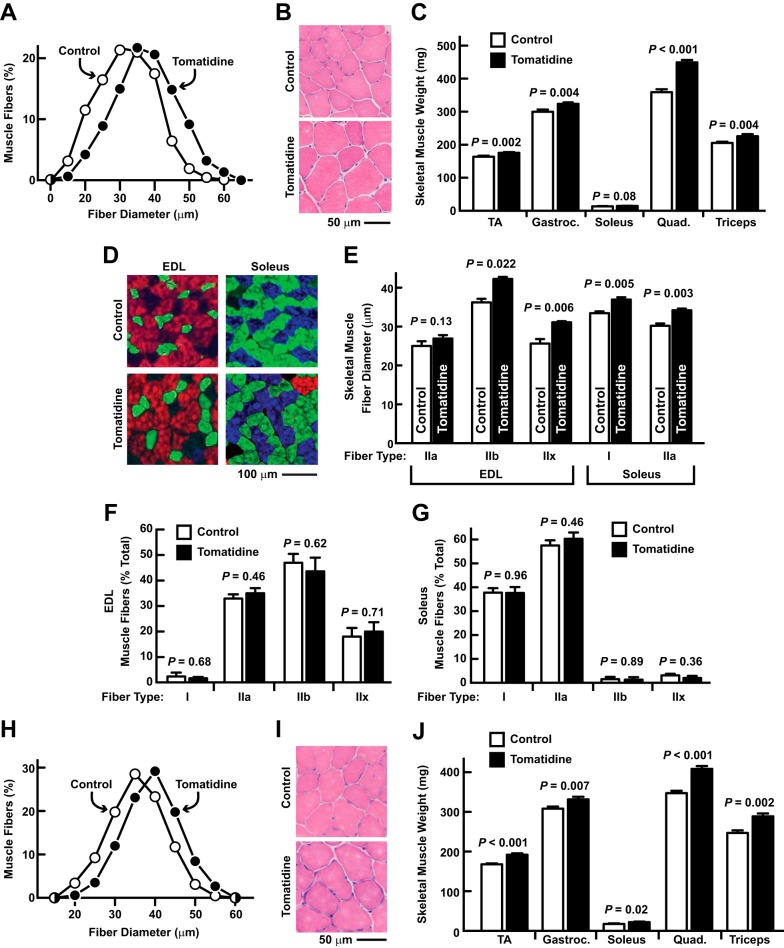
**Tomatidine stimulates skeletal muscle hypertrophy.** *A–G*, Seven-week-old mice were provided *ad libitum* access to either standard chow (control) or standard chow supplemented with 0.05% tomatidine for 5 weeks before analysis of skeletal muscle fiber size and skeletal muscle weight. *A*, skeletal muscle fiber size distributions. Data are from > 1100 fibers from 5–6 TA muscles per condition. *B*, representative H&E stains of TA cross-sections. *C*, weights of bilateral TA, gastrocnemius (*gastroc*.), soleus, quadriceps (*quad*.), and triceps muscles. Data are means ± S.E. from 16 mice per condition. *D–G*, extensor digitorum longus (*EDL*) and soleus muscles were sectioned and stained with antibodies to detect fiber type-specific myosin heavy chain (*MyHC*) isoforms. *Blue*, MyHC I; *green*, MyHC IIa; *red*, MyHC IIb; *black*, MyHC IIx. *D*, representative images. *E*, muscle fiber diameters. *F* and *G*, quantification of muscle fiber types in the extensor digitorum longus muscle (*F*) and soleus muscle (*G*). Data are means ± S.E. from 3 EDL and 3 soleus muscles per condition. *H–J*, sixty-one week old mice were provided *ad libitum* access to either standard chow (control) or standard chow supplemented with 0.05% tomatidine for 9 weeks before analysis of skeletal muscle fiber size and skeletal muscle weight. *H*, skeletal muscle fiber size distributions. Data are from > 2500 fibers from 5–6 TA muscles per condition. *I*, representative H&E stains of TA cross-sections. *J*, weights of bilateral TA, gastrocnemius (*gastroc*.), soleus, quadriceps (*quad*.) and triceps muscles. Data are means ± S.E. from 11 mice per condition. *C*, *E–G*, *J*, *p* values were determined with Student's *t* test.

Consistent with its effects on mTORC1 signaling, tomatidine significantly increased skeletal muscle fiber size (Fig. 5, *A* and *B*), and increased the weight of tibialis anterior (TA), gastrocnemius, quadriceps, and triceps muscles (Fig. 5*C*). We also detected a trend toward increased weight in the soleus muscle (Fig. 5*C*). The overall increase in skeletal muscle mass was 13.7 ± 0.0% (*p* < 0.001).

Further examination of skeletal muscle fibers revealed that tomatidine stimulated hypertrophy of glycolytic muscle fibers (types IIb and IIx) in the extensor digitorum longus muscle, and oxidative muscle fibers (types I and IIa) in the soleus muscle (Fig. 5, *D* and *E*). Tomatidine did not alter the relative amounts of type I, IIa, IIb, or IIx fibers (Fig. 5, *F* and *G*).

To determine if tomatidine also has the capacity to stimulate muscle hypertrophy in older mice that are no longer growing, we added 0.05% tomatidine to the diet of middle-aged adult mice (61 weeks old) for 9 weeks. As it had done in young mice, tomatidine induced skeletal muscle fiber hypertrophy (Fig. 5, *H* and *I*), and this was accompanied by hypertrophy of all measured skeletal muscles (TA, gastrocnemius, quadriceps, triceps and soleus; Fig. 5*J*). The overall increase in skeletal muscle mass was 14.2 ± 0.0% (*p* < 0.001).

**FIGURE 6.**
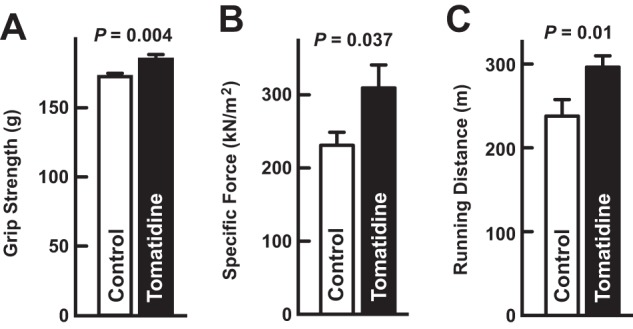
**Tomatidine improves muscle function.** Seven-week-old mice were provided *ad libitum* access to either standard chow (control) or standard chow supplemented with 0.05% tomatidine for 5 weeks before analysis of skeletal muscle function. *A*, grip strength. Data are means ± S.E. from 16 mice per condition. *B*, specific tetanic force generated by the EDL muscle *ex vivo*. Data are means ± S.E. from 4 mice per condition. *C*, distance run on an accelerating treadmill. Data are means ± S.E. from 16 mice per condition. *A–C*, *p* values were determined with Student's *t* test.

In addition to increasing skeletal muscle mass, tomatidine significantly increased grip strength *in vivo* (Fig. 6*A*), and specific muscle force *ex vivo* (Fig. 6*B*). Furthermore, tomatidine increased running distance on an accelerating treadmill (Fig. 6*C*). Taken together, these data indicate that tomatidine stimulates skeletal muscle anabolism *in vivo*, leading to muscle hypertrophy, increased strength, and improved exercise capacity.

##### Tomatidine-induced Skeletal Muscle Hypertrophy Is Accompanied by a Loss of Fat

**FIGURE 7.**
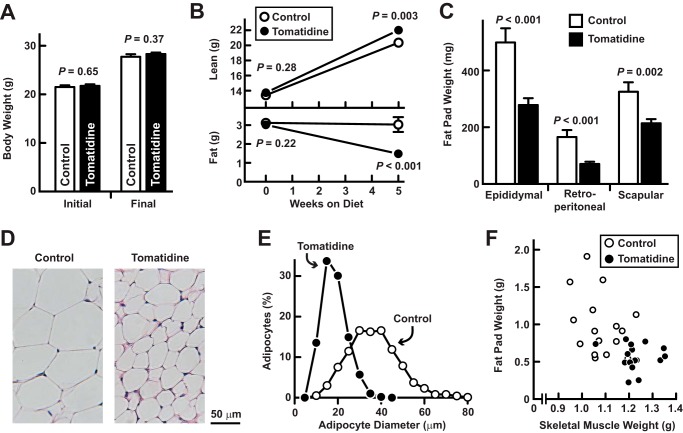
**Tomatidine-induced muscle hypertrophy is accompanied by reduced adiposity.** Seven-week-old mice were provided *ad libitum* access to either standard chow (control) or standard chow supplemented with 0.05% tomatidine for 5 weeks before analysis of body composition and adiposity. *A*, initial and final total body weights. Data are means ± S.E. from 16 mice per condition. *B*, initial and final lean and fat mass, assessed by NMR. Data are means ± S.E. from 16 mice per condition. *C*, weights of bilateral epididymal, retroperitoneal, and scapular fat pads. Data are means ± S.E. from 16 mice per condition. *A–C*, *p* values were determined with Student's *t* test. *D*, representative H&E stain of adipocytes. *E*, adipocyte size distributions. Data are from > 1400 adipocytes from 3–4 fat pads per condition. *F*, relationship between skeletal muscle weight (bilateral TA, gastrocnemius, soleus, quadriceps, and triceps) and adipose weight (bilateral epididymal, retroperitoneal, and scapular fat pads). Each data point represents one mouse.

Although tomatidine increased skeletal muscle, it did not increase total body weight (Fig. 7*A*), suggesting that tomatidine reduced the weight of another tissue. Since skeletal muscle hypertrophy is often accompanied by a loss of total body fat (7, 10, 27–30), we used nuclear magnetic resonance to analyze body composition. We found that tomatidine increased lean mass, but reduced fat mass (Fig. 7*B*). Furthermore, tomatidine significantly reduced the weights of the epididymal, retroperitoneal and scapular fat pads (Fig. 7*C*), which was explained by a reduction in adipocyte size (Fig. 7, *D* and *E*). Thus, tomatidine had two major effects on body composition, namely, increased skeletal muscle and decreased fat (Fig. 7*F*).

##### Tomatidine Reduces Skeletal Muscle Atrophy and Enhances Recovery from Skeletal Muscle Atrophy

**FIGURE 8.**
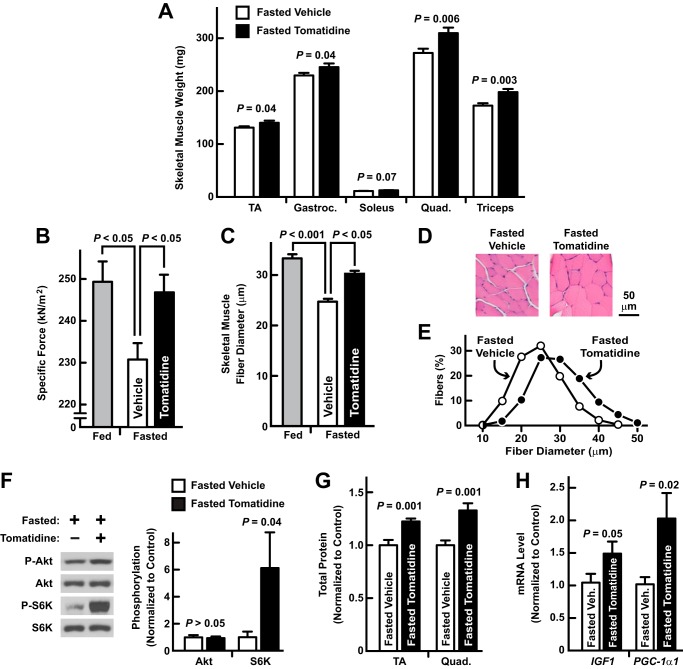
**Tomatidine reduces skeletal muscle atrophy during fasting.** *A*, mice were fasted and administered intraperitoneal injections of either vehicle (corn oil) or tomatidine (25 mg/kg) at the beginning of the fast and 12 h later. Bilateral TA, gastrocnemius (*gastroc*.), soleus, quadriceps (*quad*.) and triceps muscles were harvested after 24 h of fasting. Data are means ± S.E. from 8–9 mice per condition. *B–E*, mice were allowed *ad libitum* access to food (fed) or were fasted. Fasted mice were administered intraperitoneal injections of either vehicle or 25 mg/kg tomatidine at the beginning of the fast and 12 h later. Fed and fasted mice were harvested 24 h after the beginning of the fast. *B*, *ex vivo* EDL specific tetanic force. Data are means ± S.E. from 13–14 muscles per condition. *C*, mean TA muscle fiber diameters ± S.E. from 4–5 muscles per condition. *D*, representative H&E images of TA cross-sections from fasted mice. *E*, TA fiber size distributions from fasted mice. Data are from > 700 fibers from 4–5 muscles per condition. *F–H*, mice were fasted, and then administered intraperitoneal injections of either vehicle or 25 mg/kg tomatidine at the beginning of the fast and 12 h later. Skeletal muscles were harvested 14 h after the beginning of the fast (*F*) or 24 h after the beginning of the fast (*G–H*). *F*, immunoblot analysis of total and phosphorylated Akt and S6K. *Left*, representative immunoblots. *Right*, quantification. Data are means ± S.E. from 7 gastroc. muscles per condition. *G*, total cellular protein. Data are means ± S.E. from 5–6 TA and 5–6 quad. muscles per condition. *H*, qPCR analysis of *IGF1* and *PGC-1*α*1* mRNA levels. Data are means ± S.E. from 5–6 TA muscles per condition. *A–C*, *F–H*, *p* values were determined with Student's *t* test (*A*, *F–H*) or ANOVA with Tukey's post hoc test (*B–C*).

The strategy that led us to tomatidine, coupled with tomatidine's anabolic effects in skeletal muscle, suggested that tomatidine might have a capacity to reduce skeletal muscle atrophy. As an initial test of this hypothesis, we investigated whether tomatidine inhibits skeletal muscle atrophy during fasting. In fasted mice, intraperitoneal administration of tomatidine increased skeletal muscle mass, indicating a reduction in fasting-induced skeletal muscle atrophy (Fig. 8*A*). Tomatidine also reduced the loss of skeletal muscle specific force during fasting (Fig. 8*B*), and it decreased fasting-induced skeletal muscle fiber atrophy (Fig. 8, *C–E*). Importantly, these effects on skeletal muscle were accompanied by increased mTORC1 activity, as assessed by increased S6K phosphorylation (Fig. 8*F*), increased total cellular protein (Fig. 8*G*), and increased *IGF1* and *PGC-1*α*1* mRNAs (Fig. 8*H*). Akt phosphorylation was unchanged (Fig. 8*F*).

**FIGURE 9.**
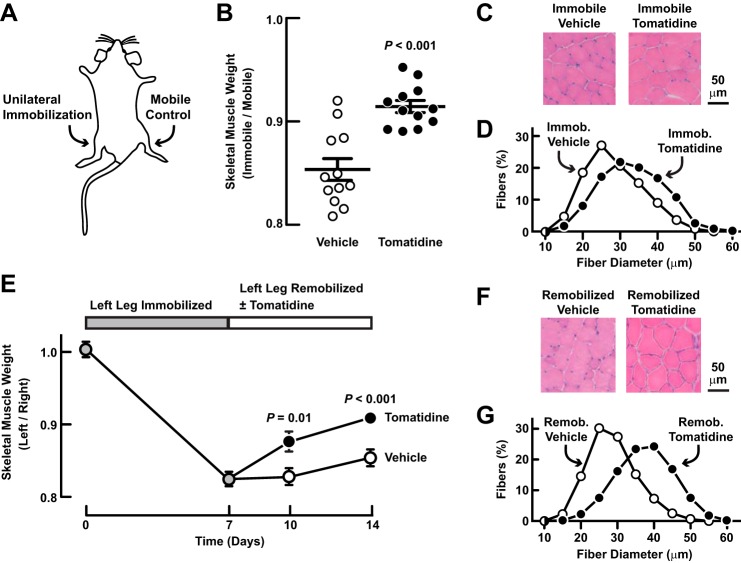
**Tomatidine reduces skeletal muscle atrophy during and after limb immobilization.** *A–D*, mice were administered intraperitoneal injections of vehicle or 25 mg/kg tomatidine every 12 h for 8 days. On day 2, unilateral hindlimb immobilization (*A*) was performed. On day 8, bilateral TA muscles were harvested for analysis. *B*, TA muscle weight. In each mouse, the weight of the immobile TA was normalized to the weight of the contralateral (mobile) TA. Each data point represents one mouse. Horizontal bars denote means ± S.E. *C*, representative H&E images of cross-sections from immobile TAs. *D*, fiber size distributions from immobile TAs. Data are from > 2500 fibers from 5 muscles per condition. *E–G*, left hindlimbs of mice were immobilized for 7 days to induce atrophy, and then remobilized for 7 days. Upon remobilization, mice were administered intraperitoneal injections of vehicle or 25 mg/kg tomatidine every 12 h. *E*, TA muscle weight. In each mouse, the weight of the left TA was normalized to the weight of the right (control) TA. Data are means ± S.E. from 4 mice on days 0 and 7 and 8 mice on days 10 and 14. *F*, representative H&E images of cross-sections from day 14 TAs. *G*, fiber size distributions from day 14 TAs. Data are from > 1400 fibers from 4 TAs per condition. *B* and *E*, *p* values were determined with Student's *t* test.

To test the effect of tomatidine in a second mouse model of skeletal muscle atrophy, we administered tomatidine to mice during unilateral hindlimb immobilization (Fig. 9*A*). In this model, muscles in the immobilized hindlimb undergo disuse skeletal muscle atrophy; the contralateral hindlimb, which remains mobile and non-atrophic, serves as an intrasubject control (8, 11, 12). Importantly, administration of tomatidine during limb immobilization reduced the loss of skeletal muscle mass (Fig. 9*B*) and decreased skeletal muscle fiber atrophy (Fig. 9, *C* and *D*). Thus, tomatidine reduced skeletal muscle atrophy in two distinct models: fasting and limb immobilization.

To determine whether tomatidine might enhance recovery from skeletal muscle atrophy, we immobilized mouse hindlimb muscles for 1 week to induce muscle atrophy, and then remobilized the hindlimb muscles in the absence or presence of tomatidine. As expected, under control conditions, skeletal muscle mass recovered very slowly during the first week of remobilization. However, tomatidine significantly enhanced the recovery of skeletal muscle mass (Fig. 9*E*) and muscle fiber size (Fig. 9, *F* and *G*). Collectively, these data indicate that tomatidine facilitates both prevention and treatment of skeletal muscle atrophy in mice.

## DISCUSSION

In the current study, we sought to discover a small molecule that might be used to treat skeletal muscle atrophy. The strategy that we took was unbiased and fundamentally different from traditional drug discovery methods because it relied on systemic effects of small molecules rather than predefined molecular targets or pathways. Interestingly, this systems-based strategy, accompanied by *in vitro* and *in vivo* testing in cultured myotubes and mouse skeletal muscle, elucidated tomatidine as a small molecule inhibitor of skeletal muscle atrophy.

Our data indicate that submicromolar concentrations of tomatidine act quickly and directly on skeletal muscle cells to stimulate mTORC1 signaling. This leads to increased protein synthesis, protein accretion, accumulation of mitochondria, induction of anabolic gene expression, and ultimately, cell growth. As a result, tomatidine limits the progression of skeletal muscle atrophy during fasting and muscle disuse, and enhances the recovery from disuse skeletal muscle atrophy. Taken together, these data suggest tomatidine may have potential as a therapeutic agent and/or lead compound for skeletal muscle atrophy in humans.

In skeletal muscle, mTORC1 signaling not only reduces muscle atrophy, but also promotes muscle hypertrophy. Thus, in addition to reducing muscle atrophy, tomatidine stimulates skeletal muscle hypertrophy. Importantly, tomatidine's hypertrophic effects are evident in both fast and slow muscle fibers, leading to increases in both muscle strength and exercise capacity. Like other interventions that stimulate skeletal muscle hypertrophy, tomatidine also decreases fat. The mechanism by which tomatidine decreases fat is not yet known. Possibilities include increased basal energy expenditure (a typical consequence of muscle hypertrophy), secretion of a muscle-derived factor that reduces fat, and/or a direct effect of tomatidine on adipocyte signaling and metabolism. Determining this mechanism and whether tomatidine reduces obesity are important areas for future investigation.

Identifying the molecular target of tomatidine in skeletal muscle is another important and challenging area for future work. Our data strongly suggest that the molecular target of tomatidine is present in both humans and mice, since tomatidine stimulates anabolism and hypertrophy in human myotubes, mouse myotubes, and mouse skeletal muscle. In addition, Connectivity Map analysis indicates that tomatidine's effects on mRNA expression in human cell lines approximate a mirror image of the changes in skeletal muscle mRNA expression that occur during skeletal muscle atrophy in humans.

An attractive feature of tomatidine is that it is found in food. Although our studies did not rigorously test for safety, we observed beneficial effects of tomatidine on body composition, strength, and exercise capacity without overt toxicity at the doses tested. Similarly, previous studies have shown that dietary supplementation with ∼0.1% tomatidine for 2 weeks is safe in pregnant and non-pregnant mice (31), and dietary supplementation with ∼0.04% tomatidine for 10 weeks reduces plasma cholesterol and atherosclerosis in ApoE-deficient mice without evidence of toxicity (32). In humans, dietary tomatidine comes from ingestion of α-tomatine, which is abundant in green tomatoes (up to 0.5 g/kg fresh weight), but typically decreases by ∼99% as tomatoes ripen (20). It appears that humans can safely consume green tomatoes as well as tomato cultivars that fail to degrade α-tomatine upon ripening (20, 33). It is also interesting that α-tomatine is higher in organically grown tomatoes compared with conventionally grown tomatoes (34). These considerations suggest that tomatidine could potentially have a favorable safety profile in humans. Nevertheless, tomatidine has not been extensively studied and comprehensive safety studies will be essential before the pharmacologic use of tomatidine is investigated in humans.

If proven to be safe, tomatidine could be investigated as a potential pharmaceutical agent or lead compound for the treatment of skeletal muscle atrophy, either as monotherapy or in combination with other therapeutic agents that may be developed. Tomatidine and/or α-tomatine could also be investigated as possible ingredients in functional foods and nutraceuticals designed to maintain muscle mass and function in persons without muscle atrophy. In addition to having potential utility in its own right, tomatidine supports the concept that systems-based methods can be used to discover small molecules that improve skeletal muscle mass, function, and metabolism. Such compounds could potentially have several beneficial uses for patients and society in general.

## Footnotes

2 The abbreviations used are:

mTORC: mammalian target of rapamycin complex
IGF: insulin-like growth factor
PGC: peroxisome proliferator-activated receptor-γ coactivator
H&E: hematoxylin and eosin
TA: tibialis anterior.
